# ROMO1 is required for mitochondrial metabolism during preimplantation embryo development in pigs

**DOI:** 10.1186/s13008-021-00076-7

**Published:** 2021-12-16

**Authors:** Dongjie Zhou, Ming-Hong Sun, Song-Hee Lee, Xiang-Shun Cui

**Affiliations:** Department of Animal Science, Chungbuk National University, Chungdae-ro 1, Seowon-Gu, Cheongju, Chungbuk 28644 Republic of Korea

**Keywords:** Apoptosis, Embryo development, Mitochondria, Porcine, ROMO1

## Abstract

**Background:**

Reactive oxygen species (ROS) modulator 1 (ROMO1) is a mitochondrial membrane protein that is essential for the regulation of mitochondrial ROS production and redox sensing. ROMO1 regulates ROS generation within cells and is involved in cellular processes, such as cell proliferation, senescence, and death. Our purpose is to investigates the impact of ROMO1 on the mitochondria during porcine embryogenesis.

**Results:**

We found that high expression of ROMO1 was associated with porcine preimplantation embryo development, indicating that ROMO1 may contribute to the progression of embryogenesis. Knockdown of ROMO1 disrupted porcine embryo development and blastocyst quality, thereby inducing ROS production and decreasing mitochondrial membrane potential. Knockdown of ROMO1 induced mitochondrial dysfunction by disrupting the balance of OPA1 isoforms to release cytochrome *c*, reduce ATP, and induce apoptosis. Meanwhile, ROMO1 overexpression showed similar effects as ROMO1 KD on the embryos. Overexpression of ROMO1 rescued the ROMO1 KD-induced defects in embryo development, mitochondrial fragmentation, and apoptosis.

**Conclusions:**

ROMO1 plays a critical role in embryo development by regulating mitochondrial morphology, function, and apoptosis in pigs.

**Supplementary Information:**

The online version contains supplementary material available at 10.1186/s13008-021-00076-7.

## Background

Mitochondria are well-known organelles that produce adenosine triphosphate (ATP), which is important for controlling cell growth, signaling, dynamic response, and apoptosis in most mammalian cells. In porcine oocytes and/or embryos, a high level of ATP production in the cytoplasm is necessary for oocyte maturation, fertilization, and early embryo development in vivo and in vitro [1]. Pig oocytes and embryos differ from those of other species in having a large quantity of endogenous lipid. Particularly, after in vitro fertilization (IVF), the zygotic stage of porcine embryos contains high levels of lipids consisting of triglycerides for energy storage [2]. Many studies have shown that ATP production-related lipid metabolism is important for early embryonic development [3, 4]. During ATP production, reactive oxygen species (ROS), such as hydrogen peroxide, superoxide, and hydroxyl radicals, are generated by oxidative phosphorylation in mitochondria [5]. Production of ROS is relative to the processes of oocyte maturation, fertilization, and embryo development in pigs [6]. Many studies have shown that ROS accumulation reduces embryonic developmental competence and blastocyst quality in pigs, cattle, and mice [7–9]. Moreover, an imbalance between free radical formation and removal can lead to oxidative stress, which can induce DNA damage and increase the expression of proapoptotic genes, leading to cell death during oocyte maturation and early embryonic development [10, 11].

Additionally, various dynamic processes are involved in stabilizing mitochondrial structure and function, including fission, fusion, mitophagy, and mitochondrial biogenesis. Mitochondrial fission and fusion are involved in maintaining mitochondrial integrity, mitochondrial function complementation, mitochondrial turnover, selective removal of mutant mitochondrial DNA, and regulation of apoptosis [12, 13]. In mammalian cells, mitochondria typically form an interconnected network, but fission events can separate a mitochondrion from the mitochondrial network to be engulfed by an autophagosome [14].

Mitochondria possess a characteristic double-membrane morphology. The inner membrane is folded into cristae, which house the oxidative phosphorylation system [15]. At the cellular level, mitochondria form a highly dynamic network that undergoes constant fission and fusion events [16, 17]. A crucial regulator of mitochondrial dynamics and structure is the Reactive oxygen species (ROS) modulator 1 (ROMO1), a mitochondrial membrane protein that is involved in mitochondrial ROS production and redox sensing in mitochondrial dynamics [18]. Upregulated ROMO1 has been reported in various cancers [19]. Furthermore, ROMO1 has been implicated in the regulation of optic atrophy protein 1 (OPA1) processing and maintenance of a proper ratio between inner mitochondrial membrane-anchored long (L-OPA1) and soluble intermembrane space-localized short (S-OPA1) for normal mitochondrial morphology [20, 21]. Excessive cleavage of OPA1 by the inner membrane-associated zinc metallopeptidase OMA1 is closely associated with the destruction of cristae structure and mitochondrial malfunction [22, 23]. Processing of OMA1 (self-cleavage of M-OMA1 and accumulation of S-OMA1) accounts for its enhanced activity towards OPA1 cleavage in response to adverse stimuli, producing redundant S-OPA1 that interferes with cristae structure and promotes cytochrome *c* release [24, 25]. Previous studies have shown that ROMO1 regulates ROS generation within cells and is involved in cellular processes, such as cell proliferation, senescence, and death [26, 27]. However, the role of ROMO1 in porcine embryogenesis has not yet been studied. In our preliminary study, we found high *ROMO1* expression after zygotic genome activation (ZGA), indicating that ROMO1 may participate in porcine embryo development.

## Results

### Expression of ROMO1 mRNA and protein during early porcine embryo development

**Fig. 1:**
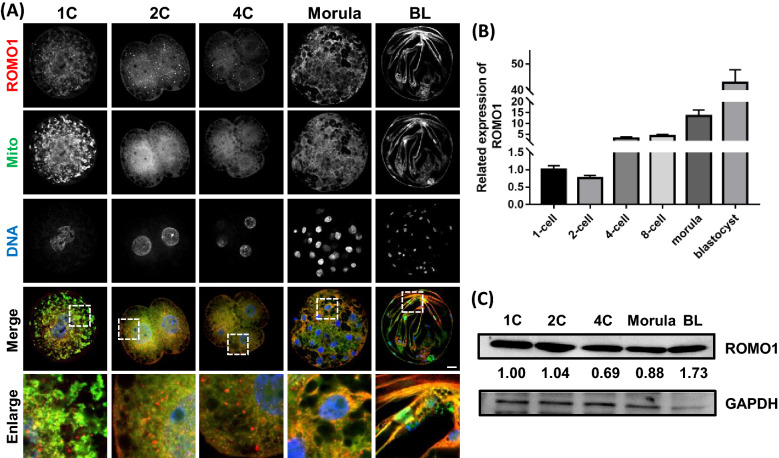
ROMO1 protein and mRNA expression during early porcine embryonic development. **A** Immunofluorescence images for ROMO1 expression at 1-cell, 2-cell, 4-cell, morula, and blastocyst stages. Scale bar, 20 μm. **B** ROMO1 mRNA expression levels were relative to their 1-cell stage expression levels. 18 s was selected as reference gene. **C** Protein levels of ROMO1 in 1-cell, 2-cell, 4-cell, morula, and blastocyst stages by western blot analysis

The expression and subcellular localization of ROMO1 in early porcine embryos are depicted in Fig. 1A. ROMO1 protein was expressed at a high level in 1-cell to blastocyst-stage embryos under normal culture conditions and co-localized with mitochondria. The mRNA expression of *ROMO1* gradually increased during embryo development until the blastocyst stage (Fig. 1B). The RNA level of ROMO1 was highly expressed from morula stage which demonstrate that ROMO1 may be crucial for embryo development after ZGA. As shown in Fig. 1C, the expression of ROMO1 protein was verified by western blotting at the 1-cell, 2-cell, 4-cell, morula, and blastocyst stages.

### Effects of ROMO1 knockdown on early porcine embryonic development

**Fig. 2:**
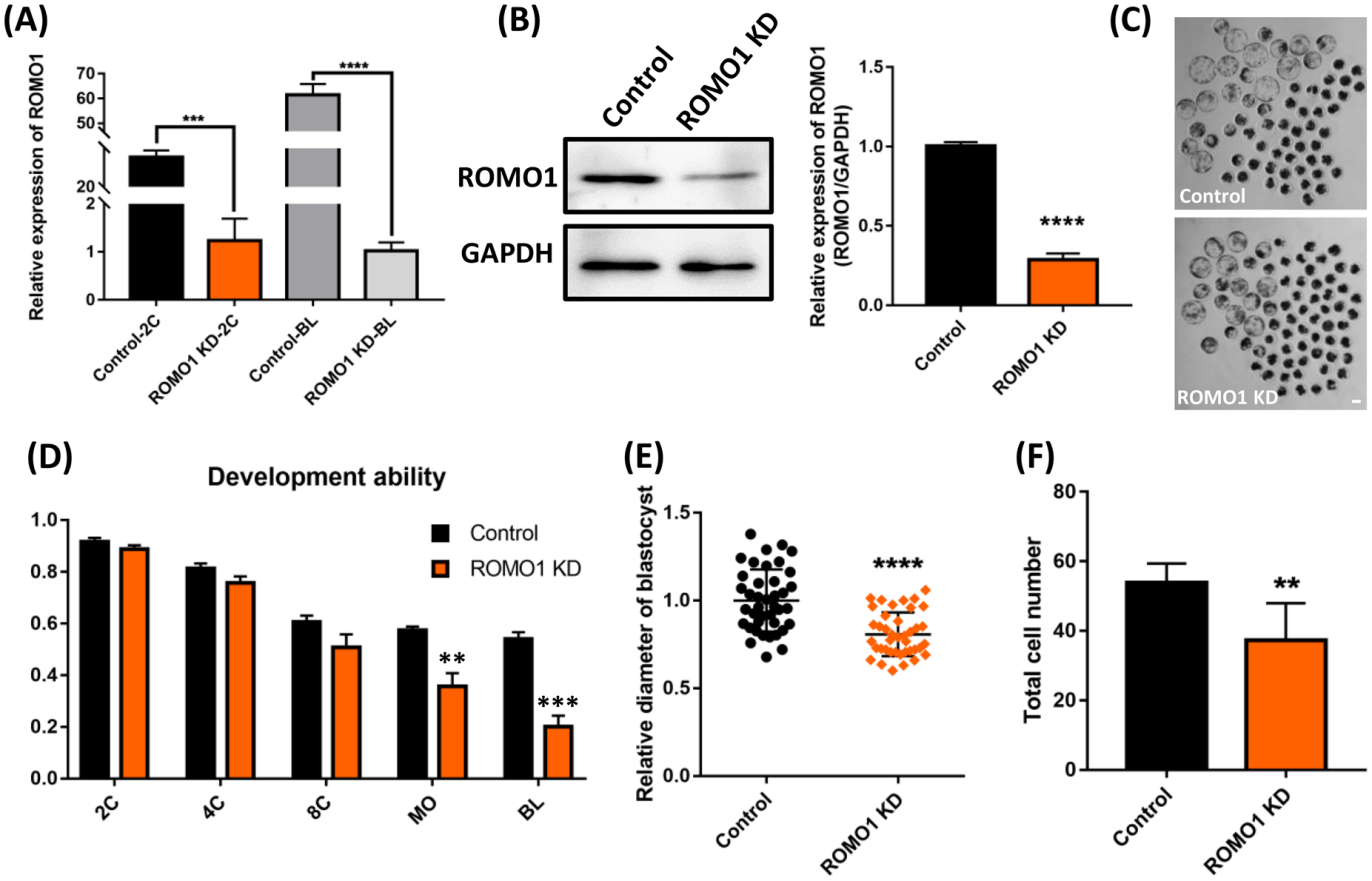
Effects of ROMO1 knockdown on early porcine embryonic development. **A** Quantitative PCR was conducted to confirm ROMO1 knockdown at the blastocyst stage. 18 s was selected as reference gene. **B** Western blotting was conducted to confirm ROMO1 knockdown at the blastocyst stage. **C** The morphology of the d-7 embryos in dsROMO1 and dsControl groups. Scale bars, 100 μm. **D** The embryo development rate from 2-cell to blastocyst stages in dsROMO1 and dsControl groups. **E** Blastocyst diameter of dsROMO1 (n = 40) and dsControl (n = 40) embryos. **F** Total cell number of dsROMO1 (n = 60) and dsControl (n = 60) embryos. **P < 0.01, ***P < 0.001 and ****P < 0.0001 indicate significant differences between treatment groups

To investigate why ROMO1 was expressed in porcine embryos during all stages and colocalized with mitochondria, *ROMO1* dsRNA (ROMO1 KD) was injected into porcine parthenotes, which were then cultured in vitro for 7 days. *ROMO1* knockdown efficiency was evaluated at the two cell and blastocyst stage. Embryos injected with enhanced green fluorescent protein dsRNA were used as the control group (Control). As shown in Fig. 2A, compared with that in the control group, *ROMO1* expression was effectively reduced by over 90% in ROMO1 KD embryos at the blastocyst stage (P < 0.001). Knockdown of ROMO1 was verified by western blotting of blastocysts (Fig. 2B, P < 0.001). Although the 2- to 8-cell cleavage rate was not affected by ROMO1 knockdown (2-cell stage, control: 91.67 ± 1.45 vs. ROMO1 KD: 88.67 ± 0.88; 4-cell stage, control: 81.33 ± 1.86 vs. ROMO1 KD: 75.67 ± 1.45; 8-cell stage, control: 60.67 ± 2.33 vs. ROMO1 KD: 50.67 ± 2.96, P > 0.05), the formation of morula and blastocyst was significantly lower in the ROMO1 KD group than in the control group (control: 57.33 ± 1.45 vs. ROMO1 KD: 35.67 ± 2.96, P < 0.01; control: 54.00 ± 2.65 vs. ROMO1 KD: 20.00 ± 2.52, P < 0.001) (Fig. 2C, D). Additionally, the blastocysts in the ROMO1 KD group were smaller than those in the control group (Fig. 2C). The diameter and total cell number of blastocysts were significantly reduced in ROMO1 KD blastocysts compared with those in the control group (194.40 ± 4.74 vs. 240.80 ± 4.75 μm, P < 0.0001; 37.38 ± 2.65 vs. 53.92 ± 5.44, P < 0.01) (Fig. 2E, F).

### ROMO1 knockdown induces mitochondrial fragmentation and dysfunction

**Fig. 3:**
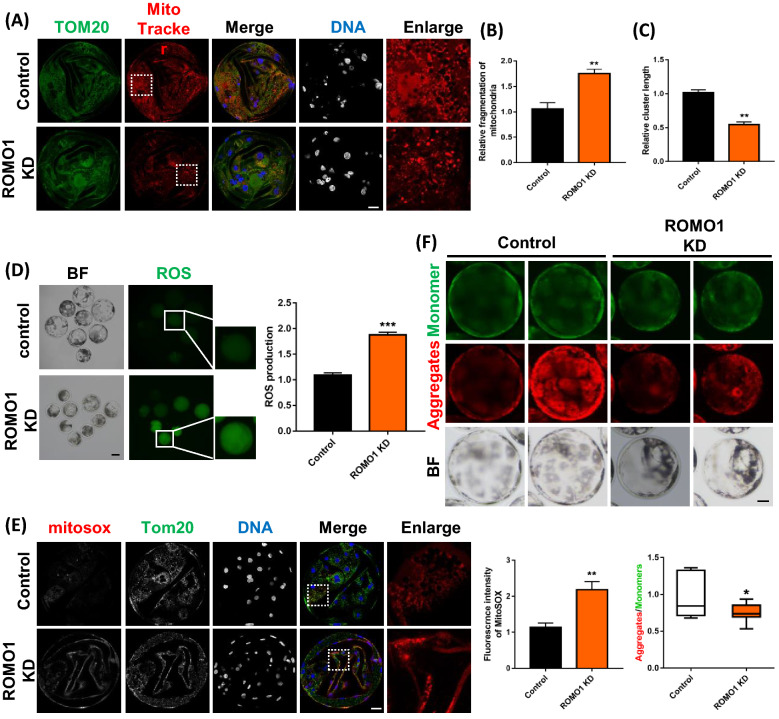
Knockdown of ROMO1 induces mitochondrial fragmentation, ROS production, and mitochondrial membrane potential depolarization in porcine embryos. **A** Representative confocal images of dsControl and dsROMO1 blastocysts showing well-preserved mitochondrial clusters in dsControl blastocysts but fragmented mitochondria clusters in dsROMO1 groups. Scale bars, 20 μm. **B**, **C** Quantification of confocal images of dsControl (n = 5) and dsROMO1 (n = 6) blastocysts showing the mitochondrial fragmentation rate and relative mitochondrial cluster length. **D** ROS levels in control and ROMO1 KD blastocysts. **E** MitoSox staining and mitochondrial ROS levels in control and ROMO1 KD blastocysts. Scale bars, 20 μm. **F** JC-1 staining and mitochondrial membrane potential in control and ROMO1 KD blastocysts. Scale bars, 20 μm. *P < 0.05, **P < 0.01, and ***P < 0.001 indicate significant differences between treatment groups

Previous studies have reported that knockdown of ROMO1 induced robust mitochondrial fragmentation in HeLa cells [20]. To investigate the effect of ROMO1 KD on mitochondrial morphology in early porcine embryos, confocal microscopy was conducted to confirm the effect of ROMO1 KD on mitochondrial dynamics after immunostaining for TOM20. As shown in Fig. 3A–C, knockdown of ROMO1 induced fragmentation of mitochondrial clusters. Total and active mitochondria were marked by TOM20 and MitoTracker Red CMORos at blastocyst stage, respectively (Fig. 3A). TOM20 staining showed that total and active mitochondria were evenly distributed in control blastocysts. However, although total mitochondria were evenly distributed in each cell, active mitochondria were clearly observed in the partial blastomeres of ROMO1 KD blastocysts. Mitochondrial cluster fragmentation induced by mitochondrial fission or inhibition of mitochondrial fusion is typically detected by confocal microscopy and measured by image J. The active mitochondria were also marked by MitoTracker Red CMORos on 4 cell stage embryos, which showed that the intensity of active mitochondria was significantly reduced by knockdown of ROMO1 (Additional file 1: Fig. S2A). Given the structural and quantitative variety of mitochondria, we investigated whether the mitochondrial function was compromised in porcine embryos after ROMO1 knockdown.

Mitochondrial dysfunction is closely related to excessive ROS generation, reduced mitochondrial membrane potential, and ATP deficiency. Accordingly, total ROS and mitochondria-derived ROS were evaluated in porcine embryos using the 29,79-dichlorodihydrofluorescein diacetate fluorescent reaction and MitoSox mitochondrial superoxide indicator, respectively. As expected, compared with control 4-cell stage embryos and blastocysts, total ROS levels in ROMO1 KD groups were significantly upregulated, respectively (Fig. 3D and Additional file 1: Fig. S2B). The level of mitochondria-derived ROS in ROMO1 KD blastocysts was also significantly increased compared with control (Fig. 3E). Furthermore, the mitochondrial membrane potential was evaluated in porcine embryos using the JC-1 fluorescent reaction. Knockdown of ROMO1 enhanced green fluorescence and reduced red fluorescence of the JC-1 dye compared with the control in both 4-cell stage embryos and blastocysts (Fig. 3F and Additional file 1: Fig. S2C). Quantification analysis showed that the ratio of fluorescence intensity (red to green) decreased in ROMO1 KD blastocysts compared with that in the control group, indicating a loss of mitochondrial membrane potential.

### ROMO1 knockdown induced apoptosis in porcine embryos

**Fig. 4:**
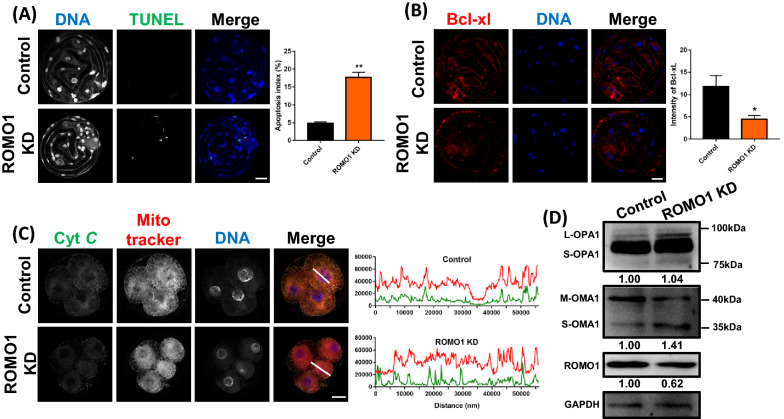
Knockdown of ROMO1 induces apoptosis in porcine embryos. **A** TUNEL assay and apoptosis index in control and ROMO1 KD blastocysts. **B** Bcl-xL levels in control and ROMO1 KD blastocysts. **C** Colocalization of cytochrome *c* and MitoTracker Red in control and ROMO1 KD 4-cell stage embryos. Scale bars, 20 μm. **D** Western blots of OPA1, OMA1, and ROMO1 in control and ROMO1 KD groups. *P < 0.05 and **P < 0.01 indicate significant differences between treatment groups

Because mitochondrial impairment and reduced total cell numbers were observed in ROMO1 KD blastocysts, we next evaluated whether apoptosis was apparent in blastocysts with ROMO1 KD by performing the TUNEL assay. As shown in Fig. 4A, TUNEL signals were significantly increased in ROMO1 KD blastocysts compared to those in the control group (P < 0.01). Besides, the Bcl-xl expression level as an anti-apoptotic protein was significantly reduced by knockdown of ROMO1 (Fig. 4B). We also performed EdU staining after ROMO1 KD which showed no significant difference between control and ROMO1 KD group (Additional file 1: Fig. S1A). Collectively, these results indicated that ROMO1 KD porcine blastocysts underwent apoptosis.

Additionally, the release of cytochrome *c* from mitochondria as a pro-apoptotic signal has been reported [28]. Therefore, we analyzed the colocalization of mitochondria and cytochrome *c* in porcine 4-cell stage and blastocyst embryos after ROMO1 knockdown. The results showed that colocalization of mitochondria and cytochrome *c* was disrupted, as detected by Pearson’s overlap coefficient (P < 0.001, Fig. 4C and Additional file 1: Fig. S1B). Previous studies have demonstrated that ROMO1 suppression leads to cleavage of OPA1 due to an imbalance of OPA1 isoforms, thus preventing OPA1 oligomerization and augmenting cristae remodeling that allows the release of cytochrome *c*. This reduced OPA1 oligomerization increases cell sensitivity to apoptotic insults [19]. Therefore, the OPA1 isoforms were visualized by western blotting. As shown in Fig. 4D, the levels of the short form of OPA1 (S-OPA1) were significantly increased in ROMO1 KD blastocysts than in the control group. In response to oxidative stress, OMA1 activity is significantly enhanced by OMA1 processing (self-cleavage of M-OMA1 and accumulation of S-OMA1), which subsequently induces OPA1 cleavage and eventually leads to mitochondrial fragmentation, cytochrome *c* release, and apoptosis [25]. To verify that ROMO1 KD regulated OPA1 processing by OMA1, the protein expression of OMA1 was visualized by western blotting (Fig. 4D). We found that S-OMA1 accumulated in embryos upon suppression of ROMO1 with decreased M-OMA1 protein levels, suggesting that ROMO1 KD promoted OMA1 cleavage during embryo development. Upregulation of mitochondrial fission prevents mitochondrial elongation induced by ROMO1 knockdown. The balance between fission and fusion is critical to mitochondrial morphology and function, and an increase in either the interconnected or fragmented state leads to disease [29].

### ROMO1 overexpression disrupts porcine embryo development by mitochondrial dysfunction

**Fig. 5:**
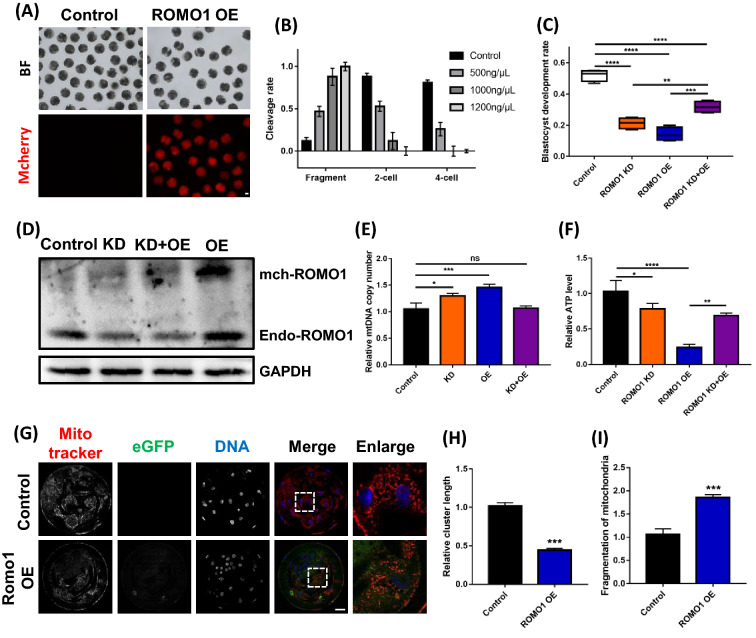
Overexpression of ROMO1 disrupts embryo development by inducing mitochondrial fragmentation. **A** The d-2 embryo morphologies of ROMO1 OE and control groups. Scale bars, 100 μm. **B** Development and fragmentation rates at 2- and 4-cell stages. **C** Development rate of blastocyst embryos in ROMO1 KD, ROMO1 OE, ROMO1 KD + OE, and control groups. **D** Western blot image of ROMO1 levels in control, ROMO1 KD, ROMO1 OE, and ROMO1 KD + OE groups. **P < 0.01 indicates significant differences between treatment groups. **E** Relative mitochondrial DNA copy number in control, ROMO1 KD, ROMO1 OE, and ROMO1 KD + OE blastocysts. **F** ATP level in control, ROMO1 KD, ROMO1 OE, and ROMO1 KD + OE blastocysts. **G** Representative confocal images of control and ROMO1 OE blastocysts showing well-preserved mitochondrial clusters in control blastocysts but fragmented mitochondria clusters in ROMO1 OE blastocysts. Scale bars, 20 μm. **H**, **I** Quantification of confocal images of control (n = 5) and ROMO1 OE (n = 5) blastocysts showing mitochondrial fragmentation rate and relative mitochondrial cluster length. *P < 0.05, **P < 0.01, and ***P < 0.001 indicate significant differences between treatment groups

To investigate whether ROMO1 regulates mitochondrial shape and morphology by mitochondrial fusion, we examined the effects of ROMO1 overexpression (ROMO1 OE) on ROMO1 KD-induced alterations in mitochondrial morphology. As shown in Fig. 5A, mCherry-ROMO1 was highly expressed in embryos, and the development rate was reduced even at 2-cell cleavage (Fig. 5B). The percentage of fragmentation, which are correlated with in vitro development and well defined [30], was significantly higher in mCherry-ROMO1 group. For the following study, 500 ng/μL of the mRNA was used for overexpression of ROMO1. However, the ROMO1 KD-induced disruption of blastocyst development rate was rescued by ROMO1 overexpression (Fig. 5C). The protein level of ROMO1 was also detected by western blotting (Fig. 5D). The mitochondrial DNA copy number control is an important aspect of mitochondrial genetics and biogenesis and is essential for normal cellular function [31]. The mtDNA copy number was detected by qPCR, which increased in both ROMO1 KD and OE embryos but was rescued in ROMO1 KD + OE embryos (Fig. 5E). ATP levels were decreased in ROMO1 OE embryos but were rescued in ROMO1 KD + OE embryos (Fig. 5F). Increased ROMO1 expression enhances cellular ROS levels and oxidative DNA damage [19, 32]. Previous studies have suggested that ROMO1 overexpression drives mitochondrial fragmentation [33]. After ROMO1 OE, the active mitochondria were stained by mitotracker red. The active mitochondria were reduced by ROMO1 OE. Mitochondrial cluster length was also reduced, and mitochondrial fragmentation was increased by ROMO1 overexpression in early porcine embryos (Fig. 5G–I). Together, these findings further support that the appropriate expression of ROMO1 promotes mitochondrial function.

### Optimal expression of ROMO1 can reduce DNA damage and apoptosis

**Fig. 6:**
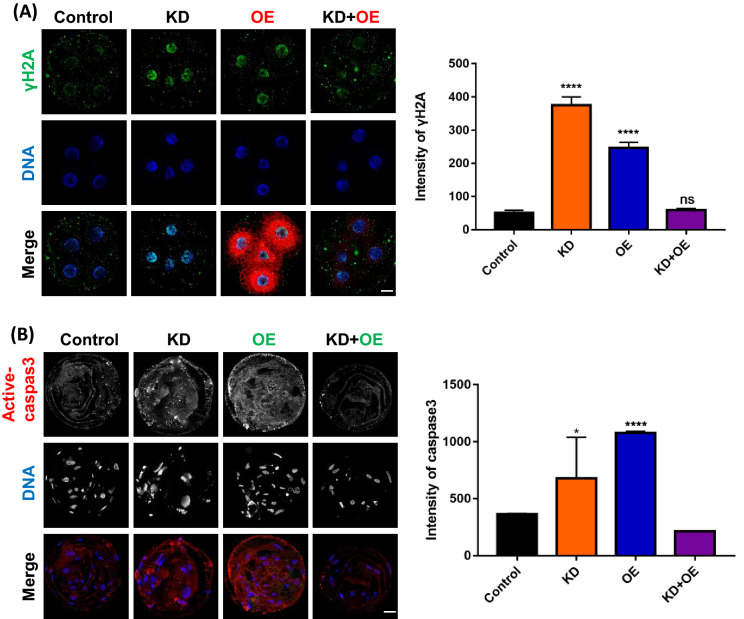
Proper expression of ROMO1 can reduce DNA damage and apoptosis. **A** Image and γH2A level in control, ROMO1 KD, ROMO1 OE and ROMO1 KD + OE 4-cell stage embryos. **B** Caspase 3 expression and intensity in control, ROMO1 KD, and ROMO1 KD + OE blastocysts. Scale bars, 20 μm. **P < 0.01, ***P < 0.001 indicate significant differences between treatment groups

Increased ROMO1 expression enhances oxidative DNA damage [19, 32]. In our study, we found that γH2A expression was significantly increased after ROMO1 KD or OE, but was rescued in the ROMO1 KD + OE group, indicating that the ROMO1 KD-induced DNA damage was rescued by overexpression of ROMO1 (Fig. 6A). Moreover, active-caspase 3 expression was detected by immunofluorescence, and high expression levels were observed in ROMO1 KD and ROMO1 OE embryos, but the active-caspase 3 levels were not significantly different between ROMO1 KD + OE and control embryos. This result suggested that the ROMO1 KD-induced apoptosis was rescued by ROMO1 overexpression (Fig. 6B).

## Discussion

Mitochondria are present in nearly all cell types, with particularly high numbers in oocytes and preimplantation embryos. Previous studies have mainly focused on the effects of environmental factors on mitochondria in mammalian oocytes and embryos, but little is known about the genetic effects of mitochondrial dynamic alterations. Several reports have demonstrated that endogenous ROS levels generated by ROMO1 are required for normal and tumor cell proliferation [18, 34]. Although excessive ROS production is harmful to cells, many studies have demonstrated that low levels of cellular ROS are essential for cell proliferation [35, 36]. An antioxidant-induced reduction in endogenous ROS causes cell cycle arrest in the G1 phase, demonstrating that steady-state ROS levels are required for entry into the S phase [37]. These reports demonstrate that serum-induced cell growth is inhibited by a decrease in endogenous ROS levels. Many reports have also demonstrated that intracellular ROS are generated by growth factors and cytokines and that they are indispensable for cell proliferation [38, 39]. ROS production required for redox signaling is mainly induced by NADPH oxidase, and various growth factors and cytokines stimulate ROS generation by activating this enzyme [40].

In the present study, we focused on the role of ROMO1, the silencing of which leads to fragmentation of the mitochondrial network due to a defect in mitochondrial fusion. ROMO1 regulates the generation of ROS in cells, linking it to cellular processes, such as proliferation, senescence, and cell death [18, 32, 34, 41]. However, the role of ROMO1 in porcine embryogenesis has not yet been studied. We found that *ROMO1* was highly expressed in blastocyst-stage embryos rather than in other stages. Therefore, we determined whether *ROMO1* knockdown disrupts porcine preimplantation embryo development. ROMO1 protein was steadily expressed during all stages and colocalized with mitochondria during porcine embryonic development. The percentage and quality of the blastocysts were impaired after *ROMO1* knockdown by dsRNA. Knockdown of *ROMO1* induced mitochondrial fragmentation with reduced cytochrome *c* levels, indicating that suppression of ROMO1 induced mitochondrial dysfunction. Interestingly, overexpression of ROMO1 showed similar defects, including mitochondrial fragmentation, embryo development impairment, apoptosis, and DNA damage in porcine early embryos.

A previous report showed that *ROMO1* siRNA transfection effectively decreased ROS levels in human cervical cancer cells, human lung carcinoma cells, and human non-transformed lung fibroblast cells [18]. However, in porcine embryos, ROS levels were increased because ROMO1 was knocked down and overexpressed. It may be that knockdown of ROMO1 impaired mitochondrial fusion. The formation of disulfide bridges within ROMO1 in response to oxidative stress is inhibitory, resulting in a fragmented mitochondrial network that allows for the containment of oxidative damage and removal of damaged mitochondria [20].

**Fig. 7:**
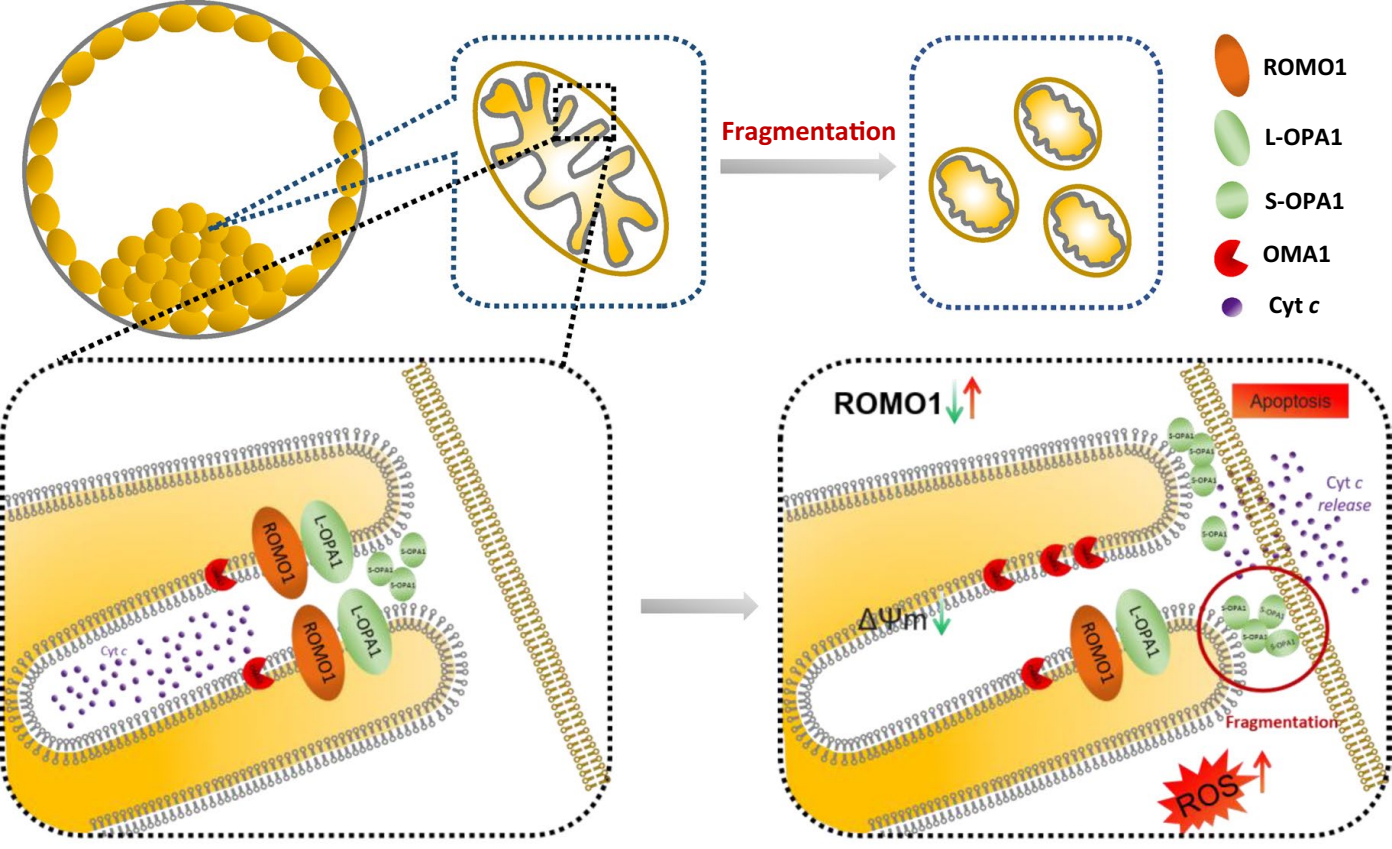
Schematic representation showing that ROMO1 controls mitochondrial morphology by promoting mitochondrial cristae formation during porcine early embryonic development L-OPA1, long-form OPA1; S-OPA1, short-form OPA1; ΔΨm, mitochondrial membrane potential; Cyt *c*, cytochrome *c*

As shown in Fig. 4, the abundance of OPA1 isoforms changes after ROMO1 knockdown, likely due to altered processing. ROMO1 and OPA1 reside in the inner mitochondrial membrane and form a complex in cells. Silencing or ablation of ROMO1 or OPA1 phenocopies each other, with respect to cristae junction integrity and inner membrane fusion [42]. Knockdown of ROMO1 induced mitochondrial fragmentation and increased ROS levels by cleavage of L-OPA1 to S-OPA1 through S-OMA1 accumulation. ROMO1 overexpression caused defects similar to knockdown, which included damage accumulation, mitochondrial depolarization, oxidative stress, and ATP deficiency. Mitochondrial depolarization-induced cytochrome *c* release caused apoptosis in porcine embryos (Fig. 7).

## Conclusions

Taken together, ROMO1 may be involved in the regulation of mitochondrial cristae dynamics through OPA1 processing and ROS generation during porcine embryo development.

## Materials and methods

### Reagents

All chemicals were purchased from MilliporeSigma (Burlington, MA, USA), unless otherwise indicated. All manipulations were performed on a heated stage adjusted to 38.5 °C, unless otherwise indicated.

### Collection of porcine oocytes and in vitro maturation

Ovaries from prepubertal gilts were collected from a local slaughterhouse (Farm Story Dodarm B&F, Umsung, Chungbuk, South Korea) and transported to the laboratory at 37 °C in saline supplemented with 75 mg/mL penicillin G and 50 mg/mL streptomycin sulfate. Follicles (3–6 mm in diameter) were aspirated using an 18‐gauge needle connected to a 10 mL disposable syringe. Cumulus-oocyte complexes were selected according to their morphological characteristics (i.e., those showing at least three layers of compact cumulus cells and evenly granulated ooplasm). Fifty cumulus-oocyte complexes were rinsed with in vitro maturation medium [TCM‐199 (11150‐059; Thermo Fisher Scientific, Waltham, MA, USA) supplemented with 0.1 g/L sodium pyruvate, 0.6 mM l‐cysteine, 10 ng/mL epidermal growth factor, 10% (v/v) porcine follicular fluid, 10 IU/mL luteinizing hormone, and 10 IU/mL follicle-stimulating hormone] and then transferred into 4-well dishes containing 500 µL of maturation medium. The medium was covered with mineral oil, and the plates were incubated at 38.5 °C in a humidified atmosphere of 5% CO_2_ for 44 h.

### Parthenogenetic activation and in vitro culture

After removing cumulus cells by repeated pipetting in 1 mg/mL hyaluronidase, denuded oocytes were parthenogenetically activated by two direct-current pulses of 120 V for 60 µs in 297 mM mannitol (pH 7.2) containing 0.1 mM CaCl_2_, 0.05 mM MgSO_4_, 0.01% polyvinyl alcohol (PVA, w/v), and 0.5 mM 4-(2-hydroxyethyl)piperazine-l-ethanesulfonic acid. These oocytes were cultured in bicarbonate-buffered porcine zygote medium 5 (PZM‐5) containing 4 mg/mL bovine serum albumin (BSA) and 7.5 µg/mL cytochalasin B for 3 h to suppress extrusion of the pseudo-second polar body. Next, the oocytes were thoroughly washed and cultured in bicarbonate‐buffered PZM‐5 supplemented with 4 mg/mL BSA in 4-well plates for 7 days at 38.5 °C and 5% CO_2_. Two‐ and four-cell cleavage rates and morula and blastocyst formation rates were examined at 24, 48, 96, and 144 h after activation, respectively. To determine the total cell number, D7 blastocysts were randomly collected and stained with 10 mg/mL Hoechst 33342 in PBS for 5 min.

### *ROMO1* double-stranded RNA preparation

To prepare *ROMO1* double-stranded RNA (dsRNA), *ROMO1* was amplified using a pair of primers (Table 1) containing the T7 promoter sequence. The purified PCR products were then used to synthesize dsRNA with the MEGAscript T7 Kit (AM1333; Thermo Fisher Scientific) according to the manufacturer’s instructions. After in vitro transcription, dsRNA was treated with DNase I and RNase A to remove the DNA template and any single-stranded RNAs, followed by purification with phenol–chloroform extraction and isopropyl alcohol precipitation. The concentration was determined by measuring the optical density at 260 nm (Nanodrop, Thermofisher, Deutsch, Germany) and adjusted to a final concentration of 1 µg/µL dsRNA aliquots was stored at − 80 °C.

**Table 1:** Summary of PCR primer

Gene	Accession	Primer sequence, 5ʹ-3ʹ	Product length (bp)	Used for
*ROMO1*	XM_001924066	F: cgttcggtgtgagacgtaga	189	dsRNA
		R: cgcattccgatcctgagaca		
*qROMO1*	XM_001924066	F: tcggcaccttttcctgtctc	186	qRT-PCR
		R: acatgggctgggactgattg		
*Mch-ROMO1*	XM_001924066	F: agatctcgagagatgcctgtggccgtg	267	mRNA
		R: atctagagcggccgcttagcatcggatgcccatc		
*eGFP-ROMO1*	XM_001924066	F: aagtccggactcgagatgcctgtggccgtg	267	mRNA
		R: cttgagctcgagttagcatcggatgcccatc		
*18S*	NR_046261	F: cgcggttctattttgttggt	219	qRT-PCR
		R: agtcggcatcgtttatggtc		

### Cloning and in vitro mRNA synthesis

The open reading frames of pig *ROMO1* (Table 1) were amplified from cDNA prepared from pig embryos. Amplified PCR products were subcloned into the pGEMHE-mCherry or eGFP-UtrCH vector, and in vitro transcription was performed using the MEGAscript T7 Kit (Thermo Fisher Scientific).

### Microinjection

For KD, OE or KD + OE experiments, 5–10  pl of *ROMO1* dsRNA, mRNA or dsRNA + mRNA was microinjected into the cytoplasm of a parthenogenetically activated oocyte respectively, using an Eppendorf Femto-Jet (Eppendorf, Hamburg, Germany) and a Nikon Diaphot Eclipse TE300 inverted microscope (Nikon, Tokyo, Japan) equipped with a Narishige MM0‐202N hydraulic 3-dimensional micromanipulator (Narishige, Amityville, NY, USA). After injection, oocytes were cultured in PZM‐5 medium. The control group was microinjected with green fluorescent protein dsRNA or empty plasmid.

### Measurement of ROS

Total ROS in blastocysts were detected using 2′,7′-dichlorodihydrofluorescein diacetate (D399; Molecular Probes, Eugene, OR, USA) as previously described [43, 44]. Briefly, blastocysts were incubated for 15 min in PBS and PVA containing 10 µM 2′,7′‐dichlorodihydrofluorescein diacetate at 37 °C. After incubation, the blastocysts were washed three times with PBS and PVA. Fluorescence signals were captured as a Tagged Image File Format (TIFF) file using a digital camera (DP72; Olympus, Tokyo, Japan) connected to a fluorescence microscope (IX70; Olympus). The MitoSox Mitochondrial Superoxide Indicator (Thermo Fisher Scientific) was used to evaluate the generation of mitochondrial ROS. Briefly, blastocysts were incubated for 30 min in PZM-5 containing 10 µM MitoSox solution at 37 °C. After incubation, blastocysts were washed three times with PBS and PVA and fixed in 3.7% paraformaldehyde for 30 min at room temperature. Total and mitochondria-derived ROS levels were quantified by analyzing the fluorescence intensity in blastocysts using ImageJ v.l.44 g software (National Institutes of Health, Bethesda, MD, USA).

### TUNEL assay

The intracellular apoptosis level of blastocysts was measured in the TUNEL assay using the In Situ Cell Death Detection Kit (11684795910; Roche, Basel, Switzerland), as previously described [45]. After washing three times with PBS and PVA, blastocysts were fixed in 3.7% paraformaldehyde for 30 min at room temperature and subsequently permeabilized by incubation in 0.5% Triton X-100 for 30 min at room temperature. The embryos were incubated with fluorescein-conjugated dUTP and terminal deoxynucleotidyl transferase enzyme for 2 h and then washed three times with PBS and PVA. Embryos were treated with 10 µg/mL Hoechst 33342 for 5 min, washed three times with PBS and PVA, and mounted onto glass slides. Images were captured using a confocal microscope (LSM 710 Meta; Zeiss, Oberkochen, Germany). The apoptosis index was calculated as the percentage of TUNEL-positive nuclei.

### Immunofluorescence and confocal microscopy

After washing three times with PBS and PVA, embryos were fixed in 3.7% paraformaldehyde for 30 min at room temperature, permeabilized with PBS and PVA containing 0.5% Triton X‐100 at room temperature for 30 min, and incubated in PBS and PVA containing 1.0% BSA at room temperature for 1 h. These embryos were incubated overnight at 4 °C with anti‐ROMO1 (1:100, 24200-1-AP; Proteintech, Cambridge, United Kingdom), anti-translocase of outer mitochondrial membrane 20 (TOM20) (1:100, F‐10, SC‐17764; Santa Cruz Biotechnology, Dallas, TX, USA), or anti-cytochrome *c* (1:100; ab110325; Abcam) diluted in blocking solution. After washing three times with PBS and PVA, the embryos were incubated at room temperature for 1 h with goat anti-rabbit IgG, rabbit anti-goat IgG, or anti-mouse IgG. The oocytes and embryos were stained with 10 µg/mL Hoechst 33342 for 5 min, washed three times with PBS and PVA, mounted onto slides, and examined under a confocal microscope (Zeiss LSM 710 Meta). Images were processed using Zen software (v.8.0; Zeiss).

### Mitochondrial membrane potential assay

The d-6 blastocysts were incubated in PZM-5 containing 2.5 µM 5,5′,6,6′‐tetrachloro-1,1′,3,3′-tetraemyl-imidacarbocyanine iodide (JC‐1) (M34152; Thermo Fisher Scientific) at 38.5 °C in 5% CO_2_ for 30 min. The membrane potential was calculated as the ratio of red fluorescence, which corresponded to activated mitochondria (J-aggregates), to green fluorescence, which corresponded to less-activated mitochondria (J-monomers) [46]. Fluorescence was visualized using an epifluorescence microscope (Nikon).

### Mitochondria and cytochrome *c* co-localization assay

To investigate the colocalization of mitochondria and cytochrome *c*, 4-cell stage embryos were incubated with 500 nM MitoTracker Red CMXRos (M7512; Thermo Fisher Scientific) at 38.5 °C for 30 min. After three washes with PZM‐5, cytochrome *c* staining was performed as described in “Immunofluorescence and confocal microscopy” section.

### Real-time RT-PCR

Embryos were collected, and mRNA was extracted from a pool of 30 embryos per group using the DynaBeads mRNA Direct Kit (61012; Thermo Fisher Scientific) according to the manufacturer’s instructions. cDNA was obtained by reverse transcription of mRNA using the Oligo (deoxythymine) 20 primer and SuperScript III Reverse Transcriptase (Thermo Fisher Scientific). The target gene *ROMO1* was amplified as follows: 95 °C for 3 min, followed by 40 cycles of 95 °C for 15 s, 60 °C for 25 s, 72 °C for 15 s, and a final extension at 72 °C for 5 min. *18S* rRNA was used as the reference gene. The primers used to amplify each gene are listed in Table 1. mRNA quantification data were analyzed using the 2^−ΔΔCt^ method [47].

### Mitochondrial DNA copy number measurements

As previously reported [48], a pool of three blastocysts was transferred to a 0.2 mL tube containing 8 µL of lysis buffer (20 mM Tris, 0.4 mg/mL proteinase K, 0.9% Nonidet‐40, and 0.9% Tween 20) at 65 °C for 30 min, followed by 95 °C for 5 min. Samples were diluted 1:25 in sterile double-distilled H_2_O before analysis. Subsequently, real-time qPCR was performed as described in the “Real-time qPCR” subsection.

### ATP measurements

ATP content was measured using a luciferin-luciferase ATP assay system with a lurninometer (CentroPro LB 962; Berthold Technologies, Bad Wildbad, Germany) according to the manufacturer’s instructions of the ATP determination kit (A22066; Molecular Probes). Briefly, 10 blastocysts were collected into a 0.2 mL centrifuge tube containing 30 µL of lysis buffer (20 mM Tris, 0.9% Nonidet-40, and 0.9% Tween 20), and these embryos were homogenized by vortexing until lysis occurred. The standard reaction solution was prepared according to the manufacturer’s instructions and was placed on ice in the dark before use. Before measurement, 5 µL samples were added to 96-well plates and equilibrated for 10 s. Subsequently, 200 µL of the standard reaction solution was added into each well, and the light signal was integrated for 10 s after a delay of 2 s. The light intensity in the control group was arbitrarily set as 1, and the light intensity in the treatment group was then measured and expressed as relative values for the control group.

### Western blot analysis

A total of 100 porcine embryos per group were placed in 1× SDS sample buffer and heated at 98 °C for 10 min. Proteins were separated by SDS-PAGE and transferred onto polyvinylidene fluoride membranes. Next, the membranes were blocked in Tris-buffered saline containing 0.1% Tween 20 and 5% skim milk for 1 h and then incubated at 4 °C overnight with anti‐ROMO1 or -GAPDH antibody. Subsequently, the membranes were incubated at room temperature for 1 h with horseradish peroxidase-conjugated goat anti-mouse IgG or goat anti-rabbit IgG (1:20,000; Santa Cruz Biotechnology). Blots were visualized using a charge-coupled device camera and UviSoft software (Uvitec, Cambridge, United Kingdom).

### Statistical analysis

Each experiment was repeated at least three times, and representative images are shown in the figures. All data were analyzed using Student’s t-test. All percentage data were subjected to arcsine transformation before statistical analysis and presented as the mean ± standard error of the mean (SEM). Significance was set at P < 0.05. All calculations were performed using SPSS software v.19 (IBM SPSS, Chicago, IL, USA). All box plots show the median (line), mean (+), and 25th and 75th percentiles (boxes), and the whiskers show the minimum to maximum values.

## Supplementary Information

**Additional file 1:** **Figure S1.** A. EdU assay and proliferative ability index in control and ROMO1 KD groups. B. Colocalization of cytochrome *c* and MitoTracker Red in control and ROMO1 KD blastocyst stage embryos. **Figure S2.** A. Representative confocal images of control, ROMO1 KD, ROMO1 OE and ROMO1 KD + OE 4-cell stage embryos for active mitochondria staining. Scale bars, 20 μm. Significant differences are represented by different capital letters (P < 0.05). B. ROS levels in control and ROMO1 KD late 4-cell stage embryos. Scale bars, 100 μm. C. JC-1 staining and mitochondrial membrane potential in control and ROMO1 KD late 4-cell stage embryos. Scale bars, 100 μm. **P < 0.01 indicate significant differences between treatment groups.

## Data Availability

All data generated or analyzed during this study are included in this published article.

## Abbreviations

ROMO1: Reactive oxygen species modulator 1
OPA1: Optic atrophy protein 1
TOM20: Translocase of outer mitochondrial membrane 20
ATP: Adenosine triphosphate
ROS: Reactive oxygen species
mtDNA: Mitochondrial DNA
IVF: In vitro fertilization
OE: Overexpression
KD: Kock-down
PVA: Polyvinyl alcohol
PZM-5: Porcine zygote medium 5
BSA: Bovine serum albumin
dsRNA: Double strand RNA
JC-1: 5,5′,6,6′‐Tetrachloro-1,1′,3,3′-tetraemyl-imidacarbocyanine iodide
