# Supplementary figures and images for: ROMO1 is required for mitochondrial metabolism during preimplantation embryo development in pigs
Source: Cell Div. 2021 Dec 16;16:7. doi: 10.1186/s13008-021-00076-7 (PMC8680150; doi:10.1186/s13008-021-00076-7)

Figure S1

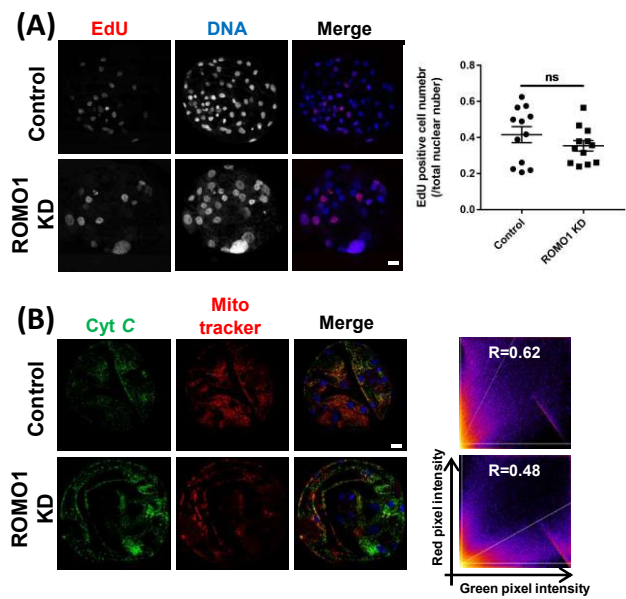

Figure S2

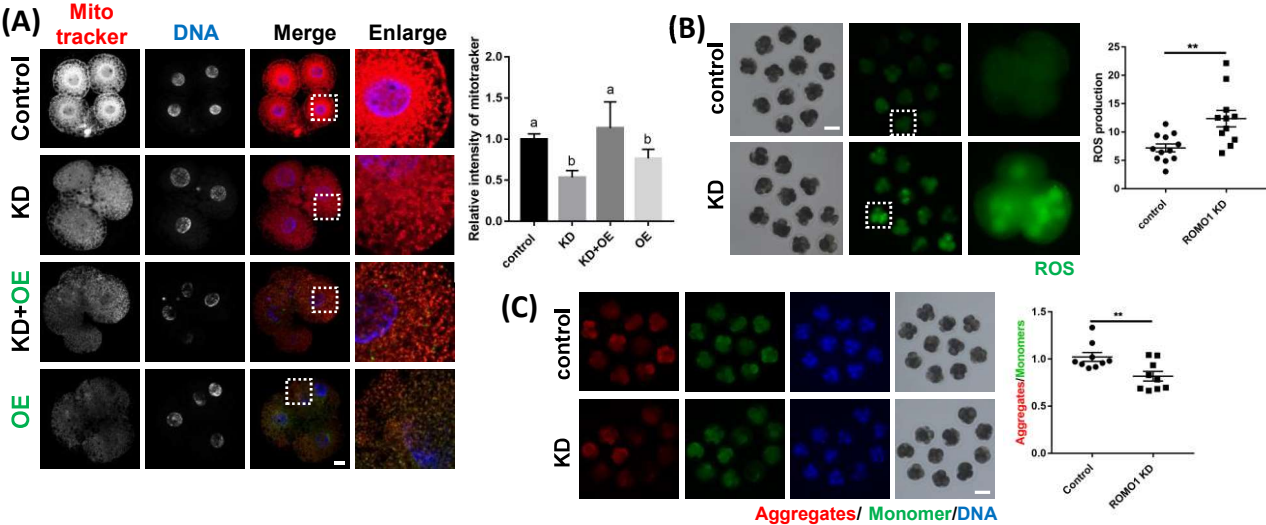

Supplement: Supplementary file 1 — Additional file 1: Figure S1. A. EdU assay and proliferative ability index in control and ROMO1 KD groups. B. Colocalization of cytochrome c and MitoTracker Red in control and ROMO1 KD blastocyst stage embryos. Figure S2. A. Representative confocal images of control, ROMO1 KD, ROMO1 OE and ROMO1 KD + OE 4-cell stage embryos for active mitochondria staining. Scale bars, 20 μm. Significant differences are represented by different capital letters (P < 0.05). B. ROS levels in control and ROMO1 KD late 4-cell stage embryos. Scale bars, 100 μm. C. JC-1 staining and mitochondrial membrane potential in control and ROMO1 KD late 4-cell stage embryos. Scale bars, 100 μm. **P < 0.01 indicate significant differences between treatment groups. [file 13008_2021_76_MOESM1_ESM.pdf]
